# Editorial for Special Issue “Newborn Screening for Sickle Cell Disease and other Haemoglobinopathies”

**DOI:** 10.3390/ijns5040036

**Published:** 2019-09-20

**Authors:** Raffaella Colombatti, Elena Cela, Jacques Elion, Stephan Lobitz

**Affiliations:** 1 Department of Child and Maternal Health, Clinic of Pediatric Hematology/Oncology, Azienda Ospedaliera-Università di Padova, 35129 Padova, Italy; 2 Department of Pediatric Oncology/Hematology, Hospital Universitario General Gregorio Marañón, Facultad de Medicina, Universidad Complutense Madrid, 28007 Madrid, Spain; elena.cela@salud.madrid.org; 3 Laboratoire d’Excellence GR-Ex, UMR_S1134, Inserm, Université Paris Diderot, Sorbonne Paris Cité, Institut National de la Transfusion Sanguine, 75015 Paris, France; jacques.elion@inserm.fr; 4 Department of Pediatric Hematology and Oncology, Gemeinschaftsklinikum Mittelrhein gGmbH, 56073 Koblenz, Germany; Stephan.Lobitz@gk.de

Sickle cell disease (SCD) is among the most common genetic disorders in the world, affecting over 300,000 newborns annually, with estimates for further increases to over 400,000 annual births within the next generation and with a wider geographical distribution of affected individuals due to global migration [1,2]. Both the World Health Organization (WHO) and the United Nations have identified SCD as a current global health burden [3,4].

The optimal care for children with SCD starts with newborn screening (NBS), which can establish a diagnosis before the onset of symptoms and allow early interventions such as prophylactic penicillin, pneumococcal immunization, screening with Transcranial Doppler ultrasound, caregiver education, and comprehensive care [5]. NBS followed by adequate comprehensive care reduce morbidity, mortality, and healthcare costs while improving the quality of life for patients.

Universal NBS is now recommended in the United States, Europe, and Brazil, although widespread implementation still needs to be achieved and many challenges remain to ensure that every child with SCD is diagnosed through NBS [6,7].

In this Special Issue on Newborn Screening for Sickle Cell Disease and Other Hemoglobinopathies (https://www.mdpi.com/journal/IJNS/special_issues/hemoglobinopathies), we have assembled a collection of review and original articles.

We have tried to cover the most widely faced challenges in the field of newborn screening for SCD: unmet needs in Europe and healthcare policy implementation as well as patient involvement and development of new diagnostic techniques.

We would like to commend the authors for the excellent reviews on the pathophysiology of SCD and thalassemia, the state of the art of NBS at a global level, and the technologies available for NBS. We would also like to praise the authors who provided original articles on specific technical topics which understanding is essential for more reliable, technically sound, and faster diagnosis.

Global diseases can be tackled only with global and coordinated efforts of different experts ranging from clinicians to technicians and basic scientists, as well as healthcare planners and the patients themselves across different countries. This Special Issue brings together a multidisciplinary global team presenting the actual situation and proposals for future developments.
